# The Human Toxome Collaboratorium: A Shared Environment for Multi-Omic Computational Collaboration within a Consortium

**DOI:** 10.3389/fphar.2015.00322

**Published:** 2016-02-17

**Authors:** Rick A. Fasani, Carolina B. Livi, Dipanwita R. Choudhury, Andre Kleensang, Mounir Bouhifd, Salil N. Pendse, Patrick D. McMullen, Melvin E. Andersen, Thomas Hartung, Michael Rosenberg

**Affiliations:** ^1^Agilent TechnologiesSanta Clara, CA, USA; ^2^Center for Alternatives to Animal Testing, Department of Environmental Health Sciences, Bloomberg School of Public Health, Johns Hopkins UniversityBaltimore, MD, USA; ^3^The Hamner Institutes for Health Sciences, Research Triangle ParkNC, USA; ^4^Center for Alternatives to Animal Testing Europe, University of KonstanzKonstanz, Germany

**Keywords:** computational toxicology, systems toxicology, cloud computing, big data, virtual machines, virtualization

## Abstract

The Human Toxome Project is part of a long-term vision to modernize toxicity testing for the 21st century. In the initial phase of the project, a consortium of six academic, commercial, and government organizations has partnered to map pathways of toxicity, using endocrine disruption as a model hazard. Experimental data is generated at multiple sites, and analyzed using a range of computational tools. While effectively gathering, managing, and analyzing the data for high-content experiments is a challenge in its own right, doing so for a growing number of -omics technologies, with larger data sets, across multiple institutions complicates the process. Interestingly, one of the most difficult, ongoing challenges has been the computational collaboration between the geographically separate institutions. Existing solutions cannot handle the growing heterogeneous data, provide a computational environment for consistent analysis, accommodate different workflows, and adapt to the constantly evolving methods and goals of a research project. To meet the needs of the project, we have created and managed The Human Toxome Collaboratorium, a shared computational environment hosted on third-party cloud services. The Collaboratorium provides a familiar virtual desktop, with a mix of commercial, open-source, and custom-built applications. It shares some of the challenges of traditional information technology, but with unique and unexpected constraints that emerge from the cloud. Here we describe the problems we faced, the current architecture of the solution, an example of its use, the major lessons we learned, and the future potential of the concept. In particular, the Collaboratorium represents a novel distribution method that could increase the reproducibility and reusability of results from similar large, multi-omic studies.

## Introduction

The Human Toxome Project is part of an ongoing effort to modernize toxicity testing with new technologies and a better understanding of toxicological mechanisms (National Research Council, 2007; Stephens et al., 2013). In particular, the project aims to map pathways of toxicity (PoT) (Hartung and McBride, 2011; Kleensang et al., 2014), the molecular network perturbations that lead to an adverse outcome as opposed to normal homeostatic change. A primary goal is to avoid prior bias, and instead deduce PoTs using abundant, readily produced multi-omic data and modern computational tools. In order to test the concept and develop the techniques (Perkel, 2012; Baker, 2013; Bouhifd et al., 2014), endocrine disruption in the human breast adenocarcinoma cell line MCF-7 (Soule et al., 1973) was chosen as a model system, and the experimental and computational work was spread across a consortium of six institutions that span the spectrum of academic, government, and commercial interests: Agilent Technologies, Brown University, Georgetown University, Johns Hopkins University (JHU) and its Center for Alternatives to Animal Testing (CAAT), the Hamner Institutes for Health Sciences, and the United States Environmental Protection Agency (EPA) (Hartung et al., 2012, 2013; Bouhifd et al., 2015).

Such a consortium is not unusual: scientific networks are expanding, and funding agencies are emphasizing collaborations (Adams, 2012, 2013). Likewise, -omics technologies continue to improve, proliferate, and produce more high-quality data at a faster rate (Marx, 2013; Khoury and Ioannidis, 2014). The intersection of these two trends presents immediate challenges, not just for toxicology, but for other fields as well. Here, we enumerate the collaborative computational challenges we encountered ourselves on the Human Toxome Project, and describe how existing solutions are inadequate. We present our own solution, The Human Toxome Collaboratorium, a shared computational environment hosted on third-party cloud services. We show how the Collaboratorium has facilitated computational collaboration, and more importantly, served to further the scientific goals of the project. Finally, we discuss the trade-offs of collaboration in the cloud, some remaining challenges, and potential future work.

## Collaborative Computational Challenges

**FIGURE 1:**
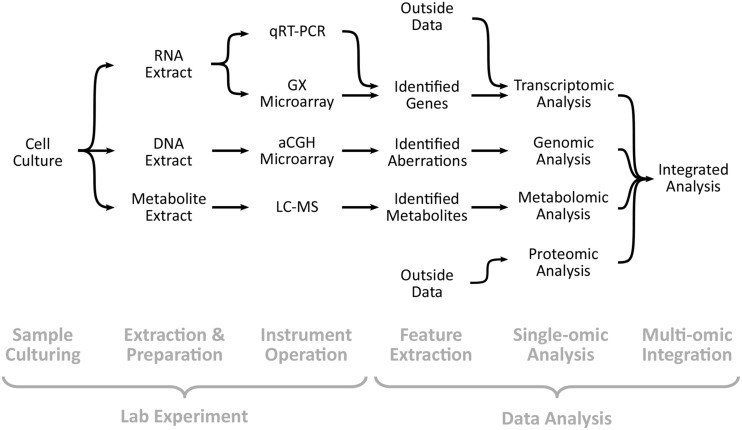
**Workflow within the Human Toxome Consortium**. Experimental data are generated via four technologies: qRT-PCR, GX microarrays, aCGH microarrays, and LC-MS (for metabolomics). Outside data from RNA-seq and LC-MS (for proteomics) have also been imported from public repositories or collaborations. Data are analyzed via a variety of tools, often producing new data to be analyzed. In many cases, the individual steps may be performed at more than one site, as shown in **Table 1**, adding to the complexity.

Within the consortium, data are generated by different laboratories at multiple sites using various technologies. A simplified view of the overall workflow is depicted in **Figure 1**, and the labs that perform each step are listed in **Table 1**. Experimental data are generated via four technologies: genomic data from array-based comparative genomic hybridization (aCGH) microarrays, transcriptomic data from quantitative reverse transcriptase-polymerase chain reaction (qRT-PCR), gene expression (GX) microarrays, and metabolomic data from liquid chromatography-mass spectrometry (LC-MS). Additional experimental data have been incorporated from collaborations and public repositories, including transcriptomic data from RNA sequencing (RNA-seq) and proteomic data from LC-MS. The experimental data are analyzed using a variety of computational tools, which themselves can produce more data for analysis. The entire workflow is complicated by the fact that different steps are performed in different labs—as shown in **Table 1**—that may be geographically distant, requiring coordination of effort. In fact, a single step may be performed in different labs depending on the context. This non-trivial experimental and analytical pipeline is likely typical of many multi-omic efforts today, more so for larger multi-omic research consortia. Within this complex multi-omic, multi-site environment, we quickly encountered many problems, particularly when it came to disseminating and analyzing data. We eventually grouped these problems into four major challenges: *sharing data*, *duplicating the computational environment*, *accommodating personal workflows*, and *adapting to change*.

**Table 1:** Overlapping responsibilities within the Human Toxome Consortium.

Results	Brown	JHU	Georgetown	CAAT	Hamner	Agilent
Cell culture	×	×				
RNA extract	×	×	×			
DNA extract		×				
Metabolite extract	×	×				
qRT-PCR	×	×	×			
GX microarray			×			
CGH microarray		×				
LC-MS				×		
Identified genes			×		×	×
Identified aberrations				×		×
Identified metabolites				×		×
Transcriptomic analysis			×	×	×	×
Genomic analysis				×		×
Metabolomic analysis				×	×	×
Proteomic analysis				×	×	×
Integrated analysis				×	×	×

*For the workflows depicted in **Figure 1**, each step may be handled by a different lab in the consortium, and the results transferred to another lab for further work. To complicate matters, a given action may be performed by more than one lab, depending on context. This table lists each step, as shown in **Figure 1**, and indicates which lab or labs are responsible*.

### Challenges Defined

*Sharing data* refers to the challenge of moving raw data, or even processed results, from one site to another. Data sets are growing ever larger in the chemical and biological sciences (Marx, 2013; Khoury and Ioannidis, 2014), and depending on the bandwidth, a modest 25 GB next-generation sequencing (NGS) data set can take more than 24 h to transfer—an actual example already encountered on the project. When multiple members want to share the same large file, the pain is multiplied. In short, email is no longer an option. In fact, all of the data should be made available to all users, and backed up in case of loss.

*Duplicating the computational environment* refers to the common problem of re-analyzing data at different sites or, simply put, viewing results sent by a colleague. As a trivial example, many people have attempted, and failed, to open a “.doc” file without the appropriate version of Microsoft Word^TM^. The problem is exacerbated in an environment with multiple technologies and multiple, sometimes undocumented, analytical pipelines. Every member of the consortium should be able to open, view, and analyze any file they might receive from another member. Moreover, members should not have to consider hardware requirements or grapple with software installation, configuration, licensing, and updating.

*Accommodating personal workflows* refers to the fact that members of the consortium—and collaborators in general—have their own distinct analytical workflows and software preferences, whether in operating systems, programming languages, libraries, frameworks, applications, folder structure, or file naming conventions. Furthermore, users have different technical abilities, so the system should be easy to use and well documented, but flexible enough to be overridden by those with particular skills.

*Adapting to change* is a common challenge in science: results can be unexpected and the questions themselves can change. As opposed to the rigid workflows one might encounter in an industrial setting, workflows in research can be more fluid. For example, on the Human Toxome Project, two-color microarrays were replaced by one-color microarrays midway through the project, requiring a change in workflow, including new reagents and protocols, as well as a reorganization of sample names and controls for each experiment. The computational tools should handle such changes in personnel, protocols, experimental design, technologies, and workflows.

### Existing Solutions

In general, there are three common types of tools that enable collaborative computation, either within an individual lab or across a larger consortium: network-connected lab workstations, shared remote servers, or web-based applications. The first solution, connected lab workstations, is arguably the most common, and many labs have already opted for it, whether in the form of desktop computers in or near the lab, or laptops carried by lab members. The solution is extremely flexible, as each collaborator can customize their computational environment to accommodate their own unique workflow. For example, one person may use Bioconductor (Gentleman et al., 2004) and R in a terminal on Linux in order to analyze microarray data; whereas a colleague may run a similar analysis using a commercial application with a graphical user interface (GUI), running on Microsoft Windows^TM^. The workstations are usually connected via a network, although file sharing tends to be *ad hoc*, most often via email or, occasionally, a shared network file system. As file sizes grow, this *ad hoc* file sharing becomes more cumbersome and difficult to manage, as each person sends large files to the others, sometimes the same large file, sometimes a slightly different version. The problem is exacerbated by distance, usually due to lower bandwidth over the Internet and the security restrictions in place at the virtual border of each institution. A more subtle problem is the need to install, configure, update, and license the required software applications on all of the workstations. Often, this is handled in an *ad hoc* fashion by the users themselves, which hides the cost but does not eliminate it. Furthermore, such *ad hoc* application management can impede collaboration when a file cannot be viewed by a recipient because the necessary application is not in place. Finally, the workstations are generally limited in computational power and take time to upgrade.

The second common solution for computational collaboration is a shared remote server, commonly accessed via network protocols and associated applications such as Secure Shell (SSH), Virtual Network Computing (VNC) (Richardson et al., 1998), or Remote Desktop Protocol (RDP). Data and software are more easily managed and shared, as the most recent versions of interest are always available on the central server. Furthermore, the central server is usually more powerful, avoiding the limitations of personal workstations. However, personal flexibility is often sacrificed in favor of centralized management. Also, a single central system tends to be even more difficult to change, or upgrade, than a personal workstation if the research requirements shift. Furthermore, accessing a shared remote server can be complicated by security restrictions at the virtual borders of labs, buildings, or institutions. It should also be noted that one of the most common methods of access, SSH, is typically performed in a terminal, or at the so-called command line, rendering the solution impractical for non-experts.

The third solution, and possibly the most common modern approach, is a web application, or web app. Several such applications exist, including Galaxy (Goecks et al., 2010), GenomeSpace^1^, the UCSC Genome Browser (Kent et al., 2002), DNAnexus^2^, Seven Bridges^3^, and Illumina BaseSpace^4^. Some are paid commercial applications; others are free and open-source. Some provide a broad range of tools; others have a narrow focus on a particular technology or workflow. Similar to a shared server, web applications simplify the sharing of data and software by centralizing the resources—indeed, the web application itself is the software—with the added benefit that web traffic is often allowed to flow freely through security checkpoints at the virtual borders of many institutions, thus simplifying remote access. Additionally, hardware concerns are often eliminated, as disk drives, RAM, and CPUs are managed behind-the-scenes. However, personal flexibility is almost completely eliminated. The platforms generally do not support legacy applications that are likely more familiar. The web application is, in fact, a new application the user must learn to do work. Also, web applications are often purpose-built, and may not adapt well to a shift in research focus.

Clearly, each of the existing solutions makes trade-offs, such as sacrificing personal flexibility for easier centralized management of the environment, that fail one or more of the collaborative challenges described in the previous section. However, the ongoing development and popularity of hardware virtualization has given rise to so-called cloud services, such as Amazon Web Services (AWS), Google Cloud Platform, and Microsoft Azure, as well as more traditional offerings from Rackspace, Linode, and Digital Ocean. These third-party services provide a foundation upon which we developed an alternate solution, with limited trade-offs, that is capable of meeting all four collaborative challenges.

## The Collaboratorium

The Human Toxome Collaboratorium is a set of virtual machines (VMs) hosted on Amazon’s cloud service, AWS. In simple terms, it is a virtual computer lab, albeit one with powerful new abilities. New machines can be created, existing machines reconfigured, and old ones discarded, in hours or sometimes minutes, all via a remote interface. As such, the Collaboratorium can adapt to changing research requirements. The primary interface for users is the familiar Microsoft Windows^TM^ desktop, accessed remotely via the Remote Desktop Protocol (RDP) from any Windows, OS X, or Linux machine. Each new VM is created based on a standard machine image, or template, that contains a full complement of relevant software applications already installed, licensed, and configured. Thus, each user begins with an identical environment, making it easier to replicate analyses and view colleagues’ results. On the other hand, users are given full administrative privileges, allowing them to customize their environment for their own preferred workflow. Finally, important data is centralized on a single shared file system that can be accessed from each VM in the Collaboratorium. The file system is hosted on a Samba server using the common Server Message Block (SMB) protocol over Amazon’s fast internal network. The underlying Linux Logical Volume Manager (LVM) provides the ability to dynamically expand the storage as needed. All of the data is backed up daily to Amazon’s Simple Storage Service (S3), a highly reliable object store. Once the data is in the Collaboratorium, it can be seen and used by every member of the Human Toxome Consortium easily. At present, the Collaboratorium serves over 40 users, using approximately 10 VMs and 5 terabytes of disk space. The base image for a new machine includes nearly 30 applications, which are listed in **Table 2**, including a mix of commercial and open-source software, along with software internally developed by members of the consortium.

**Table 2:** List of Collaboratorium software.

Application	Technology	Development
Agilent Pathway Architect 13.1.1	Integrated Biology	Commercial
Agilent OpenLAB ELN 4.2.1.0	Integrated Biology	Commercial
Hamner IDEA 1.0	Integrated Biology	Internal
Agilent Feature Extraction 11.5.1.1	Microarray	Commercial
Agilent QC Chart Tools 3.5.1.2	Microarray	Commercial
Agilent CytoGenomics 2.9.2.4	Microarray	Commercial
Agilent Genomic Workbench 7.0.4.0	Microarray	Commercial
Agilent GeneSpring GX 13.1.1	Microarray	Commercial
Agilent Mass Profiler Professional 13.1.1	MS	Commercial
Agilent MassHunter Qualitative Analysis B.06.00 SP1	MS	Commercial
Agilent MassHunter Quantitative Analysis B.06.00 SP1	MS	Commercial
Agilent PCDL Manager B.04.00 SP1	MS	Commercial
Agilent Pathways to PCDL B.05.00	MS	Commercial
Agilent Molecular Structure Correlator B.05.00	MS	Commercial
Agilent MassHunter Profinder B.06.00 SP1	MS	Commercial
Strand NGS 2.1	NGS	Commercial
Python 2.7.7	Platform	Open Source
R 3.1.1	Platform	Open Source
Oracle Java 7.0.650	Platform	Open Source
Adobe Reader XI 11.0.09	Utility	Commercial
Google Chrome 37	Utility	Open Source
Libre Office 4.3.1.2	Utility	Open Source
WinSCP 5.5.3	Utility	Open Source
7-Zip File Manager 9.20	Utility	Open Source
Notepad++ 6.6.9	Utility	Open Source
PuTTY 0.63	Utility	Open Source

*At the time of writing, the Collaboratorium included almost 30 software packages requested by members of the consortium. The list includes a mix of commercial, open source, and internally developed applications used to analyze various –omics technologies, along with general-purpose utilities and development platforms*.

Overall, the Collaboratorium handles the collaborative challenges better than the solutions previously described. The data is centralized, so although the initial upload is relatively long, subsequent processing is easier and data sharing is dramatically faster. The VMs are centralized and shared, so everyone works in the same computational environment. A remote login is required, but as a result, application installation, configuration, and licensing are simplified, and the appropriate application for a given data set is always available. Personal workflows are accommodated by installing requested software, or giving users administrative access to do so themselves, while still being monitored and managed. For unique workflows, new VMs can be created and just as easily discarded. Indeed, the virtual resources in the cloud allow for excellent adaptation over the life of the project, especially relative to the fixed resources of the typical computer lab: in the Collaboratorium, we have virtually built machines with 16 CPUs and over 100 GB RAM to handle NGS sequencing, and then quickly disposed of them after the job was done, minimizing the cost and maximizing the benefit. The Collaboratorium has its own trade-offs, but they were carefully considered and chosen to best handle the collaborative challenges we faced.

### Case Study: Analysis of Transcriptional Events Induced by Estradiol

The goal of the Human Toxome Project is to elucidate PoTs using multi–omics technologies and advanced bioinformatics. To this end, the consortium has generated extensive gene, protein, and metabolite expression datasets. To analyze the datasets, the consortium has developed bioinformatics methods, geared toward the requirements of identification and characterization of PoTs, that incorporate multiple open source and commercial software tools (see **Table 2**). Key examples of such methods are weighted correlation networks that cluster genes by network topology (Maertens et al., 2015), a sensitive method for gene enrichment analysis called Information-dependent Enrichment Analysis (IDEA) (Pendse et al., 2016), a robust method for elucidating biological response by clustering based on the responses of pathways rather than genes, as well as numerous enhancements to Agilent commercial software that were developed in response to the needs of the consortium, including metadata and correlation frameworks, support for KEGG pathways, and improved pathway visualizations. As described above, the Human Toxome Collaboratorium has been extensively used for all steps of data collection, sharing, processing, and analysis.

A critical aspect of the Human Toxome Project is establishing reproducibility of the results across different technologies, experiments, and sites, and then comparing them to previously published results. Integrating such a large, distributed, heterogeneous set of data is a bioinformatics challenge that is particularly well suited to the Collaboratorium approach. Here we present an illustrative example of integrating gene expression data from two technologies—microarrays and RNA-seq—generated by multiple labs, in order to generate a multidimensional dataset that yields a robust list of candidate genes with reproducible transcriptional changes across several independent studies in response to treatment by estrogen agonists. MCF-7 cell cultures were treated at two independent laboratories (Johns Hopkins and Brown Universities) with the estrogen receptor ligands estradiol and propyl pyrazole triol (PPT), at varying time and concentration points, and profiled on Agilent gene expression microarrays as described in Supplementary Material. Wet lab procedures and quality controls followed the manufacturer’s recommendations. The data was deposited in the Gene Expression Omnibus (GEO) (Edgar et al., 2002; Barrett et al., 2013), under GSE77244. Additionally, previously published microarray and RNA-seq profiling datasets were drawn from GEO, including GSE36586 (Dahlman-Wright et al., 2012), GSE8597 (Bourdeau et al., 2008), GSE24592 (Madak-Erdogan et al., 2011), GSE51403 (Liu et al., 2014), GSE3529 (Rae et al., 2005), GSE4006 (Chang et al., 2006), and GSE26459 (Gonzalez-Malerva et al., 2011). The data were analyzed in the Human Toxome Collaboratorium environment using Agilent GeneSpring GX for microarray analysis and Strand NGS for RNA-seq analysis as well as to align raw sequencing reads to the human genome.

**FIGURE 2:**
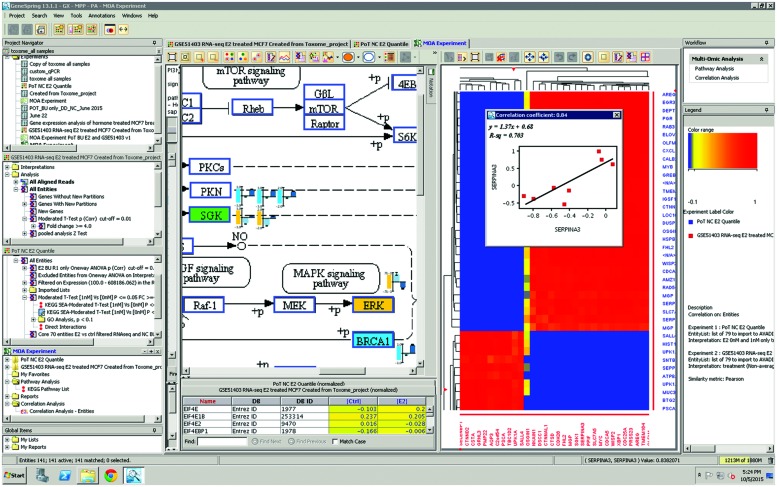
**Collaboratorium case study**. The Collaboratorium provides a central location to gather data from multiple sources, along with readily available software to analyze the data. This figure shows GeneSpring GX being used in the Collaboratorium to compare consortium-acquired gene expression microarray data to publicly available RNA-seq data, in which a significant overlap in estradiol responsive genes was found. Two multi-omic analysis (MOA) tools in the software were used for comparison: pathway analysis (left window) and cross-technology entity-entity correlation (right window).

A major challenge for identifying biomarkers of environmental effects is the perceived lack of reproducibility across studies performed by different authors. As part of the Human Toxome Project we performed a joined analysis of publicly available datasets of commonly studied breast cancer cell lines exposed to estrogen and its agonists, combined with newly generated transcript profiling data described here. Differential expression analyses comparing estrogen- and agonist-treated MCF-7 cells to their respective controls were performed taking into consideration the unique experimental designs described in publications or data repository entries. As a result, we found a small set of genes that exhibited reproducible expression changes induced by treatment with estradiol (see **Table 3**). In addition to establishing that many of the transcriptional effects of estradiol treatment of MCF-7 cells showed a common set of target genes, comparing gene expression profiling experiments from microarray and RNA-seq datasets allowed us to create detailed visualizations of similarities and differences, showing a large overlap between the responders, as shown in **Figure 2**.

**Table 3:** Common estrogen responders.

Gene Symbol	Regulation	FC (abs)					Description
		Toxome	GSE36586	GSE8597	GSE24592	GSE51403	
ATP6V0A4	Down	1.54	3.38	1.22	19.15	3.72	ATPase, H+ transporting, lysosomal V0 subunit a4
ATP8A1	Down	1.43	3.02	1.79	22.1	2.4	ATPase, aminophospholipid transporter (APLT), class I, type 8A, member 1
CDC25A	Up	1.97	2.03	2.93	2.67	2.73	Cell division cycle 25A
CSTA	Down	1.89	3.95	2.5	3.26	3.14	Cystatin A (stefin A)
CTNNAL1	Up	1.38	1.71	1.72	1.6	2.16	Catenin (cadherin-associated protein), alpha-like 1
CTNND2	Down	1.42	1.75	1.79	4.88	1.73	Catenin (cadherin-associated protein), delta 2
CTSD	Up	1.37	3.15	1.58	1.73	3.07	Cathepsin D
CXCL12	Up	6.69	6.56	3.34	17.11	11.38	Chemokine (C-X-C motif) ligand 12
DSCC1	Up	1.89	2.22	4.07	2.49	2.89	DNA replication and sister chromatid cohesion 1
EGR3	Up	24.42	16.62	2.6	8.71	8.73	Early growth response 3
ELOVL2	Up	5.98	4.28	1.35	6.25	3.16	ELOVL fatty acid elongase 2
GREB1	Up	6.61	11.77	3.12	200.34	19.21	Growth regulation by estrogen in breast cancer 1
IGFBP3	Down	8.83	2.53	2.77	6.16	3.24	Insulin-like growth factor binding protein 3
IGSF1	Up	2.2	16.71	2.33	1.02	22.26	Immunoglobulin superfamily, member 1
MYB	Up	4.98	3.4	2.35	2.19	3.61	v-myb avian myeloblastosis viral oncogene homolog
OLFM1	Up	4.01	3.45	1.61	1.37	2.76	Olfactomedin 1
PGR	Up	32.49	9.23	4.93	25.68	21.97	Progesterone receptor
PMP22	Down	1.88	2.58	1.81	2.89	2.69	Peripheral myelin protein 22
PPIF	Up	2.75	1.74	2.28	9.95	1.55	Peptidylprolyl isomerase F
PRSS23	Up	3.43	5.86	1.57	14.76	5.53	Protease, serine, 23
RAB31	Up	9.07	2.84	2.84	7.79	3.18	RAB31, member RAS oncogene family
SERPINA3	Up	7.11	4.4	1.89	1.88	1.79	Serpin peptidase inhibitor, clade A (alpha-1 antiproteinase, antitrypsin), member 3
SGK1	Up	2.95	3.39	1.96	7.63	2.86	Serum/glucocorticoid regulated kinase 1
SLC35C1	Down	2.41	1.64	1.72	1.19	1.36	Solute carrier family 35 (GDP-fucose transporter), member C1
SLC7A5	Up	2.32	3.12	1.68	4.59	2.98	Solute carrier family 7 (amino acid transporter light chain, L system), member 5
TBC1D2	Down	1.51	1.99	1.29	3	2	TBC1 domain family, member 2
TMEM164	Up	3.83	3.43	2.07	1.59	2.29	Transmembrane protein 164
UPK1A	Down	8.28	4.28	3.17	5.68	4.96	Uroplakin 1A
XBP1	Up	4.2	1.79	2.35	1.41	1.98	X-box binding protein 1

*In addition to gene expression microarray data generated by the consortium (under the column labeled Toxome), gene expression microarray datasets were imported into GeneSpring GX and RNA-seq data was aligned and quantified in Strand NGS. After normalization (quantile from microarray data and read-based for NGS), fold changes from a list of estrogen responsive genes were exported from each experiment. The 29 genes displayed in this table changed in the same direction in response to estradiol treatment in all five experiments, despite differences in doses and time points profiled. For details on previously published experiments see entries in Gene Expression Omnibus and their respective references: GSE36586 (Dahlman-Wright et al., 2012), GSE8597 (Bourdeau et al., 2008), GSE24592 (Madak-Erdogan et al., 2011), and GSE51403 (Liu et al., 2014). FC (abs) is defined as 2^∧^abs[log_*2*_(treated/control)]*.

The ability to integrate data from several sources in a single location, and perform direct data analysis of the large dataset in a computationally unconstrained, collaborative analytical environment, greatly increased our ability to interpret the data. The Human Toxome Collaboratorium enabled different groups involved in data analysis to review each other’s work in progress, exploring new ideas in real time based on input from all members of the distributed consortium. These capabilities led to exploring novel data analyses that would not have been possible in a more traditional computational environment.

### New Challenges

Although cloud-based VMs can simplify the technical administrative responsibilities, it should be made clear that the need to actively monitor and manage the machines does not disappear when working in the cloud. The Collaboratorium is still a set of machines, albeit virtual, with traditional information technology (IT) concerns. Security must be addressed, including the management of user credentials, or usernames and passwords, although security can be simplified in the cloud if access is limited through a single Internet gateway. Software must still be installed, configured, licensed, and updated as necessary, although the job is simplified with machine images, or snapshots, as the work must only be done once.

Some IT challenges subtly change in the cloud. For example, in traditional IT capacity planning and provisioning, machines and hard drives require a relatively large initial expenditure, and usually require significant lead time due to technical or budgetary reasons, which encourages over-provisioning in the short term. On the other hand, the virtual cloud hardware is generally leased by the hour, or some other unit of time, and can be reconfigured or expanded quickly. As such, the cloud encourages exact provisioning in the short term, and re-provisioning as necessary. We learned this lesson during the evolution of the Collaboratorium—we paid for VMs that sometimes sat idle and virtual disks that were partially empty. In an effort to minimize the cost, we reorganized the shared storage to consolidate large files and eliminate empty space. We implemented monitors that automatically shut down idle machines after a period of inactivity. In short, IT issues do not disappear in the cloud, and may in fact mutate.

## Discussion

In toxicology—and in other scientific fields as well—handling bigger data sets is a growing challenge, but it is just one part of the larger challenge of collaboration. Consortia like the Human Toxome Project already face the problem of sharing data, establishing consistent environments to analyze and view the data, doing so with a certain amount of personal flexibility, and still allowing for research directions to change. Thankfully, hardware virtualization provided by many third-party cloud services enables projects such as the Human Toxome Collaboratorium to meet these needs.

Since its inception, the Collaboratorium has evolved, and continues to evolve. In the future, we plan to improve the mechanisms used to import and export data to and from the Collaboratorium and further reduce storage costs by avoiding the duplication of large, unchanging files or using special storage options, such as ephemeral storage for temporary files or nearline storage for archives. We plan to further reduce the administrative overhead by improving and simplifying the methods used for account management, license management, and software upgrades. We also plan to demonstrate the concept on other cloud, or virtualization, platforms. The goal is to create and improve a portable environment that requires minimal overhead.

Indeed, our experience with the Collaboratorium suggests a tantalizing distribution mechanism for scientific results. Today, published results might include a link to the source data held in a public repository. However, the onus is on the reader to download the data, carefully attempt to reconstruct the appropriate computational environment, parse the data, repeat the analysis, and match the published results, all before any new analysis can take place. In the future, a publication could instead include a link, not to the data files, but to a live computational environment, where both the data and the necessary software are ready to be used. Such a controlled computational environment would likely increase the success rate of examining and reproducing published analyses, as well as encourage the use of the resulting methods in future work. To demonstrate the potential of the concept, a working copy of the Collaboratorium environment described in the case study, including the software, datasets, and results, is available upon request.

Not only does the Collaboratorium facilitate existing collaborations, it encourages new ones. One deterrent to new collaborative efforts, especially public–private partnerships, is the difficult question of who makes the initial capital investment. Traditionally, computational infrastructure can comprise a significant portion of the initial investment. Today, VMs require no initial investment; they only incur recurring costs. By re-using a proven architecture and existing machine images, the startup costs can be further decreased. Concomitantly, the question of physical ownership, especially when there are competing interests, is moot, as the machines are virtual. Furthermore, thanks to the virtual infrastructure, computational environments like the Collaboratorium can start small and scale quickly as needed. Effectively eliminating the up-front costs could spur new partnerships, and entirely new lines of inquiry, especially computationally intensive ones.

The Human Toxome Collaboratorium was created to solve our own collaborative needs: securely sharing data and software between multiple research labs in different organizations with different IT infrastructures, while still accommodating personal preferences and changing workflows. The solution is a shared computational environment hosted on third-party cloud services that provides a familiar virtual desktop and a ready mix of legacy and custom-built software, both commercial and open-source. The virtual infrastructure is modest, and a similar environment should serve the needs of a typical consortium, making the Collaboratorium a potential blueprint for future collaborative efforts.

## Author Contributions

RF and MR devised and implemented the Collaboratorium. CL, DC, AK, MB, SP, and PM were initial Collaboratorium users and provided feedback for improvement. RF, SP, and PM contributed custom software. RF, CL, and MR performed analysis and interpreted results in the case study. RF, CL, and MR drafted the manuscript. All authors revised the manuscript and approved the final version.

## Conflict of Interest Statement

AK, MB, SP, PM, MA, and TH declare that the research was conducted in the absence of any commercial or financial relationships that could be construed as a potential conflict of interest. RF, CL, DC, and MR are employees of Agilent Technologies, a company that manufactures and sells products used in this study.

## Footnotes

1 GenomeSpace, http://www.genomespace.org/

2 DNAnexus, http://www.dnanexus.com/

3 Seven Bridges, http://www.sbgenomics.com/

4 Illumina Basespace, http://basespace.illumina.com/
