# Supplementary material for: The Human Toxome Collaboratorium: A Shared Environment for Multi-Omic Computational Collaboration within a Consortium
Source: Front Pharmacol. 2016 Feb 17;6:322. doi: 10.3389/fphar.2015.00322 (PMC4756169; doi:10.3389/fphar.2015.00322)
Supplement: Supplementary file 1 [file Data_Sheet_1.DOCX]

Supplementary Material

The Human Toxome Collaboratorium: A Shared Environment for Multi-Omic Computational Collaboration within a Consortium

Rick A. Fasani^1^, Carolina B. Livi^1^, Dipanwita Roy Choudhury^1^, Andre Kleensang^2^, Mounir Bouhifd^2^, Salil N. Pendse^3^, Patrick D. McMullen^3^, Melvin E. Andersen^3^, Thomas Hartung^2,4^, and Michael Rosenberg^1^*

^1^Agilent Technologies, Santa Clara, CA, USA
^2^Center for Alternatives to Animal Testing, Department of Environmental Health Sciences, Bloomberg School of Public Health, Johns Hopkins University, Baltimore, MD, USA
^3^The Hamner Institutes for Health Sciences, Research Triangle Park, NC, USA
^4^Center for Alternatives to Animal Testing Europe, University of Konstanz, Konstanz, Germany

***Correspondence:** Michael Rosenberg, michael_rosenberg@agilent.com

# Supplementary Methods

## Cell Culture

MCF-7 cells were seeded at a density of 300,000 cells/well in 6-well plates and allowed to grow for 72 hours in complete growth medium composed of DMEM/F12 media supplemented with 10% fetal bovine serum (FBS, Atlanta Biologicals, Flowery Branch, GA), non-essential amino acids, 10µg/mL bovine insulin, and gentamicin. After 72 hours, cells were rinsed with PBS and placed in treatment medium composed of DMEM/F12 supplemented with 5% dextran charcoal stripped fetal bovine serum (DCC, Gemini Bio-products, Sacramento, CA, US, no. 100-119), nonessential amino acids, 6ng/mL bovine insulin, and gentamicin for 48 hours. Cells were then exposed to 0.01 nM, 0.1 nM, or 1 nM 17β-estradiol (E2, SigmaAldrich, St. Louis, MO, USA, no. E8875), 5 nM propyl pyrazole triol (PPT, Tocris, Minneapolis, MN, USA, no 1426), or vehicle control (dimethylsulfoxide , DMSO, Sigma Aldrich, no. D8418) in fresh treatment medium for 4 or 8 hours.

## RNA Preparation and Gene Expression Profiling

Total RNA from MCF-7 cells was extracted using TRIzol Reagent (Sigma Aldrich, no. T9424) according to manufacturer’s instruction, and purified using RNeasy Mini Kit (Qiagen). Purified RNA was quantified by using NanoDrop ND-1000 spectrophotometer (Thermo Scientific) and the quality of RNA was analyzed by using Agilent Bioanalyzer (Agilent). cRNA probes were synthesized and Cy3 labeled using Agilent LowInput QuickAmp Labeling Kit (Agilent) from 100 ng of total RNA. After purification, RNA probe concentration and dye incorporation were measured using NanoDrop ND-1000 spectrophotometer (Thermo Scientific). Probes were hybridized to Agilent SurePrint G3 human whole genome 8x60K microarray (Agilent) following manufacture’s protocol. Microarrays were scanned with an Agilent G2505C microarray scanner (Agilent) using protocol Agilent G3_GX_1 color, scan region 61x21.6mm, resolution 3 µm, TIFF 20 bit, R PMT 100%, and G PMT 100%. Images were processed with Agilent Feature Extraction 11.5.1.1 (Agilent) using protocol GE1_1105_Oct12 and grid template 028004_D_F_20140813. Bioinformatics analyses including quantile normalization, filtering, statistical analysis, correlation analysis, and pathway contextualization were performed in GeneSpring GX 13.1 (Agilent). E2-treated and PPT-treated samples were combined with their respective controls and normalized separately. Quantile normalized expression values of treated MCF-7 cells were compared to their respective controls using unpaired t-test with Benjamini-Hochberg correction. Responders were identified using 0.05 p-value and 2-fold change cut-offs unless otherwise noted.
